# Review of Contemporary Self-Assembled Systems for the Controlled Delivery of Therapeutics in Medicine

**DOI:** 10.3390/nano11020278

**Published:** 2021-01-21

**Authors:** Laura L. Osorno, Alyssa N. Brandley, Daniel E. Maldonado, Alex Yiantsos, Robert J. Mosley, Mark E. Byrne

**Affiliations:** Biomimetic & Biohybrid Materials, Biomedical Devices, & Drug Delivery Laboratories, Department of Biomedical Engineering, Rowan University, Glassboro, NJ 08028, USA

**Keywords:** self-assembly, amphiphilic molecules, lyophobic, lyophilic

## Abstract

The novel and unique design of self-assembled micro and nanostructures can be tailored and controlled through the deep understanding of the self-assembly behavior of amphiphilic molecules. The most commonly known amphiphilic molecules are surfactants, phospholipids, and block copolymers. These molecules present a dual attraction in aqueous solutions that lead to the formation of structures like micelles, hydrogels, and liposomes. These structures can respond to external stimuli and can be further modified making them ideal for specific, targeted medical needs and localized drug delivery treatments. Biodegradability, biocompatibility, drug protection, drug bioavailability, and improved patient compliance are among the most important benefits of these self-assembled structures for drug delivery purposes. Furthermore, there are numerous FDA-approved biomaterials with self-assembling properties that can help shorten the approval pathway of efficient platforms, allowing them to reach the therapeutic market faster. This review focuses on providing a thorough description of the current use of self-assembled micelles, hydrogels, and vesicles (polymersomes/liposomes) for the extended and controlled release of therapeutics, with relevant medical applications. FDA-approved polymers, as well as clinically and commercially available nanoplatforms, are described throughout the paper.

## 1. Introduction

The self-assembly of biomaterials at the micro- and nano-scale provides fascinating structures that help innovate medical treatments. As a result, they provide a novel route to help improve the patients’ quality of life. For example, self-assembled platforms are able to decrease the off-target effects of toxic therapeutics and increase patient compliance through dosage schedules [1,2]. Self-assembly, also known as self-organization, is a spontaneous technique used to fabricate systems currently used in drug delivery and tissue engineering. This technique, based on naturally occurring mechanisms, is becoming more attractive because of the accessibility, reproducibility, and low fabrication cost. Self-assembled polymeric drug delivery systems have been used since 1970 and their applications have been continuously growing [3,4]. These systems, most notably in the medical field, offer high flexibility, stability, tunable molecular architectures, and enhanced drug retention, as well as biocompatibility, degradability, and improved patient compliance [5,6].

The principles governing self-assembly are derived from colloid science, polymer science and supramolecular chemistry, in which thermodynamic stability is the main driving force [7,8,9,10]. Self-assembly is a phenomenon observed in amphiphilic molecules, which have a “built-in” affinity for two types of environments: Lyophilic (solvent-loving) and lyophobic (solvent-hating) [11,12]. The dual affinity comes from the covalent linking of two or more joining parts, each with different chemical characteristics. The most common amphiphilic molecules include surfactants, phospholipids, and block copolymers [11]. When these molecules are mixed with certain solvents and/or their physicochemical properties are tuned (e.g., composition, shape, surface properties), the different components self-organize to form stable structures, such as, micelles, emulsions, liposomes/vesicles, hydrogels, solid lipid nanoparticles, and polymersomes [11,12,13,14,15,16]. Recently, reviews have been published which also highlight the novelty of these nanostructures in the drug delivery field [16,17,18]. There are other novel self-assembled systems such as amphiphilic cyclodextrin and amphiphilic macrocyclic (i.e., calixarenes) for controlled drug delivery which are not covered in this review, but are featured on the cited references [19,20,21]. 

This review focuses on the most recent uses of self-assembled structures for the extended and controlled delivery of therapeutics. It involves self-assembled structures such as micelles, physically crosslinked hydrogels, and vesicles, with emphasis on the self-assembly mechanism of block copolymers in aqueous solutions. Relevant medical applications of these systems are also discussed. Furthermore, ongoing clinical trials and commercially available products that employ these self-assembled structures are also reported.

## 2. Amphiphilic Molecules—The Building Blocks of Self-Assembled Systems

### 2.1. Surfactants and Phospholipids

Surfactants, or surface-active molecules, are the building blocks of several self-assembled systems that have been extensively studied since the 20th century. These exotic molecules touch our everyday lives in countless ways. For instance, approximately 60% of these amphiphilic molecules are used in detergents and cleaning products, while the other 40% are used in decorative painting and coating products, electronic inks, paper, food, personal care, plastics, textiles, and pharmaceutics [22]. In drug delivery, surfactants can be used to facilitate controlled drug loading and release rate, as well as protection against drug degradation and toxicity [14,23,24,25,26].

**Figure 1:**
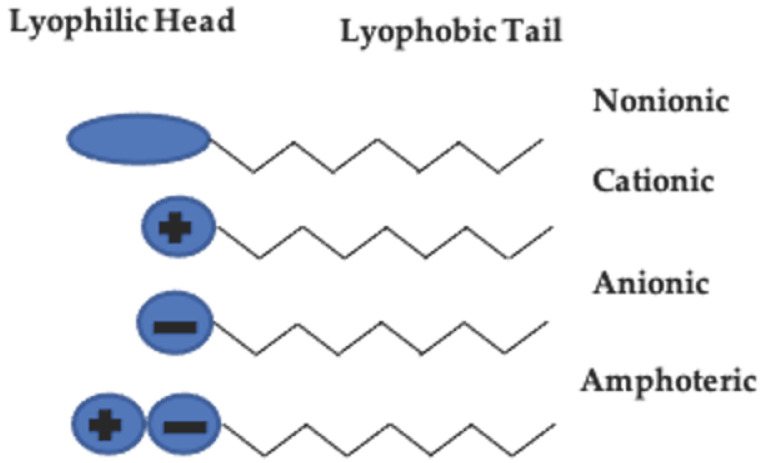
Basic molecular structure of surfactants. These molecules have dual affinities in aqueous environments, allowing them to self-assemble into different structures at the nano- and macro-scale.

Surfactants and phospholipids are amphiphilic molecules. This means that they have a dual affinity for two types of environments. Surfactants consist of a hydrophobic (water-hating) tail and a hydrophilic (water-loving) head (Figure 1). The molecular structure of these molecules is dependent on the physical and chemical properties of both the head group (size, charge, and chemical reactivity) and the nonpolar tail group (length, size, and saturation). Surfactant self-assembly is dictated by the surfactant molecular structure, surfactant concentration, solution temperature, pH, and electrolyte strength. Table 1 shows examples of different surfactants used in drug delivery.

Surfactant molecules in aqueous solutions start to self-assemble when there are enough molecules present. This point is known as the critical micelle concentration (CMC). As the surfactant concentration increases equal or higher than the CMC, the unassociated compounds aggregate due to the optimization of surface activity properties [26]. The self-assembly behavior is attributed to van der Waals forces, hydrogen bonding, hydrophobic interactions, and/or screened electrostatic interactions. These types of weak forces reduce the free energy of surfactants, allowing for enthalpic gain in solvation due to hydrogen bond formation. Additionally, the second main driving force is the hydrophobic effect, attributing the gain in entropy of water [11,26,27,28,29].

In drug delivery, nonionic surfactants are most frequently used due to their lower sensitivity to charged particles in the solvent and higher sensitivity to temperature changes [11,26,28]. Nonionic surfactants can increase bioavailability by increasing drug solubility and enhancing permeability. They are the main type of surface-active agents in ocular delivery systems because of their advantages in compatibility, stability, and toxicity. These agents are less toxic, less reactive, and less irritating to the ocular surface and they are more stable since they maintain a stable physiological pH when in solution. Carmignani et al. reported a tyloxapol, Cremophor EL, and Poloxamer 108 micellar tropicamide eye formulation that resulted in a more stable formulation compared to the commercially available eye drop version with a pH between 4.4–5.0, causing irritation [30]. Recent advantages with nonionic surfactants have involved topical dosing of ocular drugs to treat inflammation, allergies, and/or ocular hypertension [31]. Jim Jiao described the use of polyoxyethylated nonionic surfactants like polysorbates, tyloxapol, and poloxamers for topical delivery of ophthalmic drugs. A polysorbate 80 micellar solution eye drop for hydrocortisone delivery was evaluated to treat eye inflammation in rabbits. The micellar formulation led to higher hydrocortisone tissue concentrations than a non-micelle based formulation [32]. Alyami et al. described the use of surfactant-based thin films for the release of pilocarpine HCl for about 24 h. It was observed that the formulation containing Span 60 with larger vesicle diameter (overall < 500 nm) allowed for a slower release rate compared to other surfactants [33].

### 2.2. Amphiphilic Block Copolymers

In drug delivery, the use of water-soluble amphiphilic block copolymers has been of interest for the past 30 years due to their biodegradability, thermodynamic stability, phase behavior, drug solubility, and sensitivity to external stimuli such as pH and temperature. These types of copolymers have been used since 1968, with some of the first being poly(N-isopropylacrylamide) (pNIPAAM) [4,58,59], ethylene oxide—(EO), and propylene oxide—(PPO) based copolymers [11,58] as temperature-sensitive systems. 

**Figure 2:**
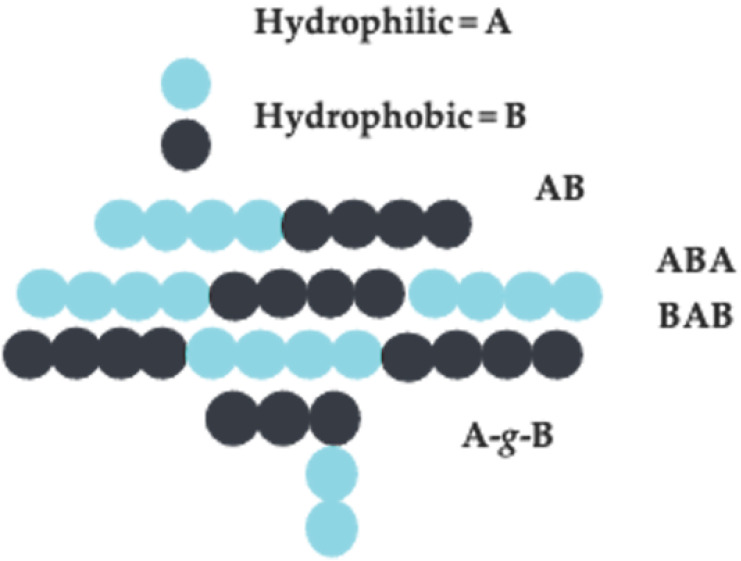
Amphiphilic block copolymers. Compounds made of identical or different types of homopolymer blocks that are covalently linked. Block copolymers of the form AB are known as diblock copolymers, of the form ABA or BAB are known as triblock copolymers, and of the form A-*g*-B or B-*g*-A are known as graft block copolymers.

Amphiphilic block copolymers are macromolecular compounds made of identical or different types of homopolymer blocks that are covalently linked together. Block copolymers are classified based on the monomer placement along the backbone, which can either be random, alternating, graft, or block-structured. Block copolymers of the types AB, ABA, BAB, and graft copolymers, where A is a hydrophilic group and B is a hydrophobic group, are widely used in drug delivery applications (see Figure 2) [11]. These types of block copolymers behave like surfactants in water and can be further classified as polyions (charged polymers) or nonionic (uncharged polymers) [23,60]. The hydrophobic block (B) is immiscible in aqueous solutions, leading to organized self-assembly of the copolymers [11,16,17,61,62]. 

Block copolymers used in drug delivery can be either synthetic or natural-based polymers. Synthetic polymers like poly(vinyl alcohol) (PVA) and poly(acrylic acid) (PAA) have been reported to enhance the mechanical moduli of natural polymers, such as collagen [63]. Poly(ethylene glycol) (PEG) has been widely used since the 1970s [64] to promote mucoadhesion [65], stabilize proteins [66], alter drug pharmacokinetics [67], and increase the lifetime of drugs inside the human body through a process known as PEGylation [68,69,70]. Poly(lactide)—(PLA), poly(ethylene oxide)—(PEO), poly(e-caprolactone)—(PCL), and poly(amino acids)-based copolymers have promising biomedical applications in drug delivery due to their structural, mechanical, and biodegradability properties [16,71,72,73,74,75,76,77,78]. On the other hand, natural polymers, or polysaccharides, possess good biocompatibility properties, making them attractive for tissue engineering scaffold applications [79]. Natural-based polymers, like polysaccharides, show stereoregular structures as they adopt supramolecular organization in response to temperature and ionic concentration [2,80,81]. For instance, chitosan, a polycation, has been used for gene delivery due to its strong affinity to DNA, forming microspheres via complex coacervation [82]. Furthermore, hyaluronic acid and its derivatives have been modified to control the degradation and release rates in drug delivery systems [83,84,85]. Lastly, alginate gels, PEG-modified gelatin nanoparticles, and albumin microspheres have been shown to incorporate macromolecules such as TGF-B1 [86,87], DNA [88], and insulin [89,90], respectively.

## 3. General Thermodynamics Self-Assembly of Block Copolymers in Aqueous Solutions

**Figure 3:**
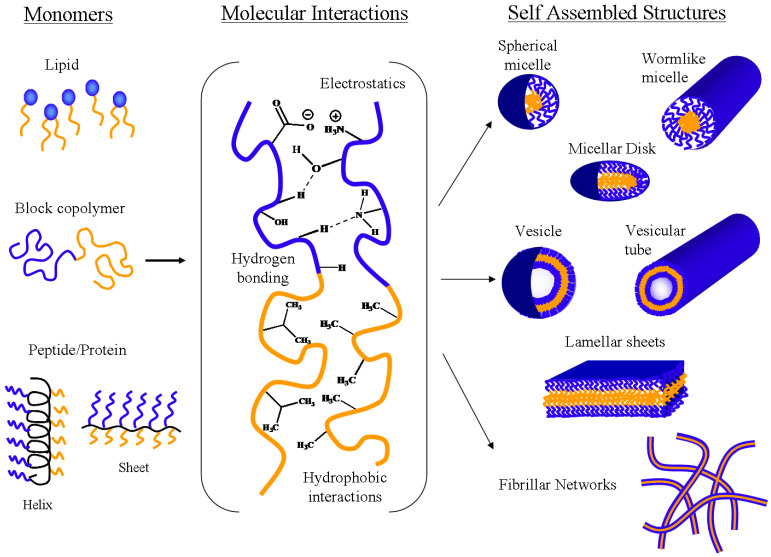
Most common self-assembled structures made from surfactants, block copolymers, and/or peptides/proteins (Reproduced with permission from [2]; Copyright Elsevier, 2020).

Block copolymers in aqueous solutions self-assemble into a variety of structures with well-defined morphology, size, and stability to deliver drug into degradative environments [91]. Figure 3 shows the most common block copolymer self-assembled structures that have been reported [11,16,17,27,61,62]. These structures tend to be regularly distributed throughout the bulk, forming long-range order and cubic arrays of spheres and hexagonally-packed cylinders [61]. This self-assembly phenomenon can be tuned and controlled by modifying the size, length, charge, and composition of the individual block copolymers, as well as the solution concentration, hydrophobic-hydrophilic balance, and overall molecular weight of the polymer [16,23,92]. A hydrophobic/hydrophilic balanced is needed for self-assembly to take place. According to Won et al., the modulation of the weight fractions (Fw) of the hydrophilic block dictates the final architecture of block copolymer nanocarriers. For instance, spherical micelles form when Fw is between 55–70% and vesicles or polymerosomes form when Fw is between 20–40% [16,93].

The self-assembly of nonionic amphiphilic block copolymers is initiated once the copolymer is dissolved in a selective solvent that is thermodynamically favorable for one block and unfavorable for the other. The main driving force is the hydrophobic effect, which attempts to minimize the unstable thermodynamic contact between nonpolar groups and water. In this matter, the water-to-polar attractions are stronger than the water-to-nonpolar attractions (van der Waals). Therefore, water molecules reorganize around the nonpolar groups in order to maximize the amount of hydrogen bonds with other water molecules (non-polar group dehydration). The exposed nonpolar functional groups, nonpolar groups in the backbone chain, temperature, pH, and presence of electrolytes in solution all influence the strength of hydrophobic interactions, as well as the polymer conformation. Increments in temperature strengthen the hydrophobic interactions as the entropic contribution of water is increased. In extreme pH and high salt concentrations, the organization of water molecules around nonpolar groups is disrupted, directly affecting self-assembly of the copolymer [14].

Polyions, for example, self-assemble in aqueous media through electrostatic interactions (driving force) forming 3-dimensional macromolecular structures known as polyelectrolyte complexes (PECs) [94]. Other intermolecular interactions, such as hydrogen bonding, hydrophobic, charge-transfer, and van der Waals interactions, can play a significant role in the formation of PECs [95]. The strength of the electrostatic interactions within PECs depends on the charge density of the polymer, conformation and composition of block copolymers, polymer molecular weight, concentration of polyelectrolyte solution, mixing ratio, and ionic strength of the solution [95]. In aqueous solutions, polyelectrolytes tend to adopt different conformations due to the dissociation of counterions from the polyions or an increase in entropy by the release of these. For example, we direct the reader to water soluble PECs; complex precipitates; or quasi-soluble PECs [14,23,95,96,97]. The final conformation structure is determined by the polyion in excess that charges the PEC surface and prevents macroscopic precipitation [95]. In drug delivery, these complexes are relevant for the storage and release of drugs and proteins, and the active substance is released either by solution equilibration, ion exchange mechanisms, charge interaction and slow decomplexation, or breakdown and dissolution of the complex [98]. Velk et al., investigated the interaction of lysozyme diffusing through poly(L-lysine)/hyaluronic acid (PLL/HA) multilayer films with pore size of approximately 10 nm. They found that diffusion coefficient of the protein is related to the extent of the change of the charge of the film, leading them to conclude that a charge balance in the film governs protein-film interaction [99]. Shiraishi et al. prepared PECs of sodium tripolyphosphate and chitosan for the controlled release indomethacin. The effects of the molecular weight of chitosan hydrolysates on the release and absorption rates were examined. It was found that with increasing molecular weight of the polyelectrolytes, the release rate was decreased [100].

Novel techniques for the self-assembly of polyions, such as layer-by-layer assembly, are being used and applied for the extended and controlled release of therapeutics. Layer-by-layer (LbL) self-assembly consists of the alternative deposition of two more polyvalent materials on a surface driven by electrostatic interactions [101,102]. Non-covalent interactions like hydrogen bonding, van der Waals forces, and hydrophobic interactions may influence the morphology, stability, thickness, molecule depositions, and permeation properties of the formed structures. The preparation method is robust and simple, and it does not need precise stoichiometry, sophisticated equipment, or complicated chemical reactions [101,102]. In drug delivery, the number of layers of the structure determines the extent of diffusion resistance and encapsulated core dissolution. Using LbL self-assembled structures can prevent burst release, a major challenge of the drug delivery field. Drug loaded nanogels (<200 nm) presented in a paper from Tan et al. were coated with alternating layers of poly(allylamine hydrochlorine) (PAH) and poly(sodium 4-styrenesulfonate) (PSS). Burst release decreased with the increasing number of self-assembled layers from 90% of drug release to 40% of drug released in the same time frame, three hours [103].

## 4. Self-Assembled Structures and Their Applications in Drug Delivery

The use of drug delivery vehicles is beneficial for many pharmaceutical agents that systemically circulate through the body. This includes small hydrophobic agents that are administered intravenously or parenterally, such as paclitaxel (cancer treatment), levonorgestrel (female birth control), and morphine (pain relief). Administering drug parenterally is associated with poor retention at the site of delivery, while for systemic delivery short circulation half-life and other side effects are problematic [104]. These small, hydrophobic drugs often have limited water solubility, poor bioavailability, and/or short half-lives. These drugs as well as other molecular forms of therapeutics can be easily encapsulated or retained to be properly delivered using self-assembled structures with hydrophobic interiors and/or hydrophilic exteriors as their nanocarriers. In 2020, Yetisgin et al. and Mitchell et al. published detailed reviews about the importance of engineering and designing disease specific drug delivery nanocarriers [18,105]. In the case of protein- or peptide-based therapeutics, delivery from a nanocarrier can greatly improve the drug’s efficacy [2]. Chan et al. showed that Basulin^®^ is the only once-a-day insulin release platform with a proof of efficacy in human clinical trials [106]. Table 2 lists current FDA approved polymers used as self-assembled platforms, like micelles, hydrogels, and vesicles (polymersomes/liposomes), and their applications in therapeutic delivery.

### 4.1. Micelles

A micelle is an amphiphilic aggregate of surfactants, or amphiphilic block copolymers, in which the hydrophilic blocks are in contact with water or a polar solvent to form the corona, while the hydrophobic blocks form the micelle core. Micelles are typically between 10 and 100 nm in size and form spontaneously in aqueous solutions. Micelle structure and size depends on parameters such as the size of the hydrophobic domain, size of the polar group, ionic strength, concentration of surfactant in solution, temperature, and pH. Micellar structures include sphere, lamellar, worm-like, and disk-shaped structures (Refer to Figure 3) [16,17,28].

Polymeric micelles were first introduced in the 1990s as drug delivery systems. They are formed via self-assembly of diblock or triblock copolymers in aqueous solvents. Block copolymer micelles can be classified into two main categories based on the intermolecular forces that drive self-assembly: Nonionic (hydrophobic interactions) and polyion complex (electrostatic interactions) [123].

Block copolymer micelles are large enough to avoid renal excretion, thus making them ideal for targeted drug delivery to specific tissues/cells through modification of their outer surface [124,125,126]. Micelles’ surface functionalization and drug conjugation has been controlled through the versatile modification of the materials’ functional groups [127]. Micelles can easily carry highly efficacious, non-polar therapeutics inside the hydrophobic micelle core, and polar therapeutics in the micelle outer shell, with the encapsulation mechanism being dependent on the drug physicochemical properties [128]. Typically, amphiphilic copolymers used for drug delivery applications have a hydrophobic segment made of a polyester or a poly(amino acid) as the center [129]. Conversely, PEG, PEO, or mPEG are commonly used to form the hydrophilic shell. PEG chains induce steric hindrance on the particle surface, allowing the micelles to circulate in the body for extended periods of time by preventing the adsorption of plasma proteins and avoiding recognition by the mononuclear phagocytic system (MPS). Other polymers that have been used as hydrophilic shells are poly(trimethylene carbonate), poly(N-vinyl pyrrolidone), poly(vinyl alcohol), polysorbate, and Vitamin E d-alpha-tocopheril polyethylene glycol 1000 succinate (TPGS or Vitamin E TPGS) [130,131,132]. Table 2 lists other FDA-approved polymers used in the formation of micelles for drug delivery purposes. 

Alami-Milani et al. used PCL-*b*-PEG-*b*-PCL micelles of approximate size of 30 nm for the sustained delivery of dexamethasone with the goal enhancing bioavailability of poorly water-soluble drugs for ocular applications. The polymeric formulations showed sustained release of dexamethasone with a low burst release [35]. In 2018, Chen et al. designed poly(γ-L-glutamic acid) (PGA) and L-phenylalanine ethyl ester (PAE) amphiphilic block copolymer which formed micelles in aqueous solution between 100 and 200 nm. The micellar platform exhibited excellent drug-retaining capabilities in physiological conditions. Micelles with smaller diameter showed extended release of cisplatin (up to 20 days) and higher tumor tissue retention [108]. Yuan et al. prepared poly(ethylenimine)-graft-poly(N-vinylpyrrolidone) (PEI-*g*-PVP) graft copolymer to self-assemble into stable, spherical shaped micelles in aqueous medium of size around 142 nm. Folic acid (FA), loaded via electrostatic interactions, was used as the model drug to test the micelles’ feasibility as drug carriers. The drug release kinetics can be controlled by changing the pH of the release medium. The slowest release rate of FA was obtained at pH 1.7, due to the larger number of hydrogen bonding interactions between the polar groups in FA and the copolymer [112]. Wang et al. constructed di-block copolymer micelles containing boronic esters and N-isopropyl acrylamide (<150 nm). The release of the cargo was stimulated by esterase and reactive oxygen species (ROS). Their doxorubicin loading capacity was calculated to be 6.99 wt.% and the entrapment efficiency was 76.99%. The release rate was controlled by the presence of ROS and esterase, with higher release rates for 3 days when both stimuli were present [133]. Triolo et al. designed ethylenediamine and lipoic acid-based micelles (PHEA-PEG_2000_-EDA-LA) for the delivery of beclomethasone dipropionate (BTD) for pulmonary administration to treat lung diseases. The authors showed sustained release of BTD from the 200 nm micelles under the chosen experimental conditions for a time period of 24 h. Approximately, 56% of the loaded cargo was released from the micelles while 90% of the drug was released from the non-commercial drug suspension [134].

The physical and morphological behaviors of the polymeric micelles can be tailored through engineering and synthesizing block copolymers [135]. Ultrafast ring-opening polymerization reaction can be used to control the size and polydispersity of the micelles. Lv et al. reported the preparation of spherical unimolecular micelles through dendritic polyamine-initiated ultrafast ring opening polymerization of N-carboxyanhydrides. The degree of polymerization was controlled, obtaining unimolecular micelles with predictable sizes. The particles were all below 240 nm, all with very low polydispersity indexes [136]. Razavi et al. used spiropyran-initiated atom transfer radical polymerization to create a novel category of light- and temperature-responsive micelles. The size of the poly(N-isopropylacrylamide) (PNIPAM)/poly(methyl methacrylate) (PMMA) micelles was influenced by temperature and UV light irradiation. The average particle size varies between 450 and 510 nm. The authors were able to further control the size through further arrangement and manipulation of the block copolymers in the formulation [137].

The ability to adjust the period of time over which the drug is released, the capability of triggering drug release at a specific time, and the ability of targeting specific tissues and cells can all be controlled through surface functionalization, which includes the addition of auxiliary agents or the modification of the total copolymer molecular weight and block length ratios [124,125,126,135,138,139,140,141]. For example, in oncology, ligand functionalization of the micelle surface increases uptake of the drug loaded micelles by the tumor cells. Yoo and Park functionalized doxorubicin-loaded PEG-PLGA micelles (<100 nm) with folic acid, which showed enhancement in cell uptake over non-targeted micelles in vitro [142]. Farokhzad et al. used an RNA aptamer on PEG-PLA micelles (250 nm–2 μm) to target the prostate-specific membrane antigen (PSMA) on prostate tumor cells. The aptamer functionalized micelles showed 77-fold increase in binding, compared to the control group [143]. Jeong et al. demonstrated that the release rate of Adriamycin was slower in poly(gamma-benzyl L-glutamate)-*b*-poly(ethylene oxide) (PBLG-*b*-PEO) micelles (<150 nm) with longer hydrophobic chain length (PBLG) [144]. 

Other means of simple, non-expensive, and versatile methods for surface functionalization in drug delivery are layer-by-layer self-assembly. Wang et al. prepared calcium carbonate particles that were modified with alternating polyelectrolytes, poly-L-ornithine (PLO) and fucoidan by LbL self-assembly. (1–2 μm). Doxorubicin loading capacity was about 79%, and it was slowly released, with 35% of the cargo released in 6 days [145]. The same group fabricated a novel nanoparticle-based drug delivery system using two polyelectrolytes, poly-allylamine hydrochloride (PAH) and fucoidan. The nanoparticles (<200 nm) were loaded with methotrexate (MTX). The release profile was biphasic, and the release rate was controlled through the electrostatic interactions between the polyelectrolytes and the drug [146]. Fan et al. designed PLGA-(PLO/fucoidan)_4_ core-shell nanoparticles with a mean size of 170 nm. The functionalized nanoparticles showed controlled and sustained release profiles of doxorubicin. About 40% of the loaded cargo was released from the functionalized nanoparticles, and 60% of the loaded cargo was released from the PLGA nanoparticles in three days [101].

Recently, block copolymer micelles have gained considerable attention as versatile nanocarriers for cancer treatment [147]. Their ability to increase circulation time, reduce toxicity, improve therapeutic index, and control and target drug release allows cancer treatments to become localized. Poorly water-soluble therapeutics are easily encapsulated in the hydrophobic core of the polymeric micelles, helping to prevent the drug’s non-specific distribution, low therapeutic index, low bioavailability, and embolism [1,148,149]. Li et al. demonstrated that poly(ethylene glycol)-phosphatidylethanolamine (PEG-*b*-PE) micelles are excellent solubilizers for the anticancer drug camptothecin (CPT). The 15 nm size loaded micelles were stable upon storage and firmly retained the loaded cargo [148]. Vitamin K3 (VK3) and 1,8-diazabicyclo[5,4,0]undec-7-ene (DBU) were co-encapsulated into 15–45 nm poly(ethylene glycol)-*b*-bdistearoyl phophoethanolamine (PEG-DSPE) micelles by Want et al. The loaded micelles showed synergistic anticancer effects against both murine and human cancer cell in vitro [149]. Koopaei et al. used PEG-PLGA micelles (<250 nm) for sustained release of docetaxel for 12 days. The authors optimized the loading capacity using the desirability function which is used for the concurrent determination of the optimum setting that can determine optimum performance levels. The release profile exhibited a biphasic pattern, with an initial burst release during the first day [150]. Moreover, dual therapy imaging and treatment for cancer are being investigated. Zhen et al. designed a co-delivery system based on poly(5-mthyl-5-propargyl-1,3-dioxan-2-one) (PMPMC), in which the fluorophore functions as a photosensitizer for image-guided photodynamic therapy and paclitaxel is the chemotherapeutic. The authors showed release of paclitaxel for two days from particles less than 160 nm in size. The release rate from these novel micelles was controlled through the use of reducing agents. Furthermore, the authors showed tumor imaging in-vivo after the nanocarriers were injected [151].

Cytotoxicity is the first step to test a drug delivery system’s suitability to be used in the human body. Costamagna et al. developed PEG-pDA (pentacosadiynoic) micelles to test the in-vitro uptake and overall response of macrophages. Polymerized micelles showed more cell viability than non-polymerized micelles. About 60% viability was observed for high concentration of micelles at a time-dependent manner (>1 mg/mL). Moreover, results showed that the micelles did not cause an increased in cell death compared to the untreated groups [152]. Wang et al. used folate-mediated Pluronic-based micelles for the solubilization and delivery of paclitaxel to improve tumor cellular uptake. The folate-mediated micelles (<200 nm) showed improved cytotoxicity levels due to their increased cellular internalization. The authors stated that such micellar system can be beneficial for the treatment of solid tumors [153]. Su et al. engineered lecithin-stabilized micelles of less than 200 nm for the delivery of docetaxel as a drug delivery system to potentially prostate cancer. The authors showed that the designed lecithin-stabalized micellar formulations showed higher cytotoxicity compared to cultures with free drug. However, the micellar formulations showed effective retardation of tumor growth. Further studies are taking place to improve the cytotoxicity levels [154].

### 4.2. Hydrogels

Hydrogels are cross-linked polymer network structures that can absorb significant amounts of water [155,156,157,158,159,160] and not dissolve, making them similar to natural tissue. Synthetic hydrogels were introduced by Wichterle and Lim in the late 1950s [161,162] and have been extensively used in the biomedical and pharmaceutical fields as drug carriers since the 1980s [15,155,157,158,160,163,164]. Properties such as biocompatibility, tunability, deformability/elasticity, ease of formulation, and porosity allow for the controlled release of proteins and peptides-based drugs [157]. Stimuli-responsive, also known as smart hydrogels or nanogels, exhibit a reversible swelling behavior which allows their release kinetics to be modulated in response to external stimuli, such as, pH, temperature, ionic strength, and electric field, among other external stimuli [15,157,158,160,165].

There are two general types of hydrogels: Physical and chemical. Physical gels are formed via physical processes, such as association, aggregation, crystallization, complexation, and/or noncovalent interactions. On the other hand, chemical gels form via chemical processes/reaction, such as chemical covalent cross-linking. Physical hydrogels are more flexible and reversible due to conformational changes, while chemical hydrogels are permanent due to configurational changes [15,160,166]. 

Physically crosslinked or self-assembled hydrogels constitute a class of soft hydrogels, also known as in situ forming hydrogels or nanogels. These can spontaneously self-assemble in response to external stimuli [2,167]. These types of gels are extremely important in the medical field as they can be biodegradable and allow for localized, controlled drug delivery, easy administration, surgery-free implantation and degradation, and improved patient compliance. The microstructure of these gels consists of orderly crosslinked block copolymer self-assembled structures. This allows for high encapsulation of large drug payloads, as well as for controlled drug release kinetics [139,168]. The most extensively investigated biodegradable polymers that self-assemble into hydrogels are PLA, PGA, PLGA, PPO, PEG, PCL, and poly(amino acids) [169,170]. Table 2 lists some biopolymers that have been widely used for self-assembled hydrogels as drug delivery platforms. 

**Figure 4:**
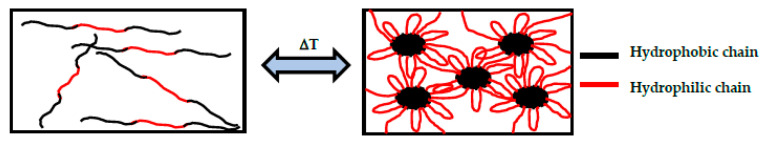
Thermo-responsive hydrogels. These amphiphilic block copolymers undergo a sol-to-gel phase transition in aqueous media. At temperatures lower than the critical gelation temperature (CGT), these polymeric solutions are homogenous and injectable. Whereas at temperatures higher than the CGT, a 3-dimensional structure of bridged micelles, dominated by hydrophobic interactions, is formed.

Temperature-responsive, injectable hydrogels have also been widely studied. They are characterized by the presence of ethyl, methyl, or propyl groups, and undergo a sol-to-gel phase transition, also known as reverse thermal gelation [62,167,168,170]. These types of aqueous polymeric formulations are homogeneous solutions at or below room temperature and undergo gelation at physiologically-relevant temperatures (Figure 4). 

The reversible gelation process is an entropic-based, hierarchical process, during which micellization is the first step. This causes an increase in the entropy of the polymeric system (Δ*S*) leading to negative free energy (Δ*G*) as per Gibbs Free Energy equation [167]. This process is then completed by the entanglement of the micelles, leading to a structured network [171].

(1) ΔG= ΔH−T(ΔS)

In addition to temperature, temperature-responsive hydrogels also respond to changes in pH, ionic strength, and/or the presence of substrates in the physiological system [167,172]. Drug release kinetics can be controlled by the polymer glass transition, the swelling degree of the polymer matrix, the diffusivity of the drug, and the degradation rate of the polymeric hydrogel. Moreover, both the hydrogel’s erosion/degradation mechanism and the mass transport of the drug within the matrix are governed by the structural morphology of the polymeric gel, which is affected by the polymer concentration, polymer type, drug aggregation inside the gel, or presence of additives within the formulation [15,173,174,175]. Zeng et al. studied the release kinetics of salbutamol from poloxamer hydrogels modified with other polymeric additives, satiaxane, and sodium chloride. They observed that for all hydrogels, solution concentration influenced the release rate. Hydrogels that did not gel at 37 °C showed the fastest release rate. Also, the addition of sodium chloride accelerated drug release. Although sodium chloride enhances the gelation and gel strength of poloxamer hydrogels, the release rate increased due to increased water uptake rate into the gel (osmotic effect), which reduced the dissolution time of the gel. As a consequence, the release rate was accelerated [173]. 

One of the most common thermosensitive controlled-release delivery systems is known as ReGel^TM^, which is comprised of PLGA and PEG triblock copolymer (PLGA-*b*-PEG-*b*-PLGA). Different ReGel formulations have been designed to deliver small hydrophobic molecules, peptides, and proteins [119,176,177,178]. For example, OncoGel^TM^ is a ReGel formulation that has been designed to deliver paclitaxel to solid tumors for a period of 6 weeks [119]. In 2013, the Institute of Bioengineering and Nanotechnology, along with IBM Research, developed a new, non-toxic hydrogel capable of shrinking breast cancer tumors faster than existing therapies. This Vitamin E-incorporated hydrogel is simply injected under the skin to sustainably release anti-cancer drugs over several weeks. This treatment has proven to be a better alternative, as it reduces the need for multiple injections for drug administration, improving patient compliance [179]. RADA 16-I is a novel, injectable, self-assembled peptide hydrogel that has shown promise as a suitable three-dimensional cell culture material. In 2019, Cheng et al. used RADA16-I hydrogel for the slow and controlled release of the one of the most widely studied chemokines that plays a crucial role in neural remodeling and survival (CXCL12). Controlled and dose-dependent release during 7 days was achieved, and cell migration was induced after 12 h [180].

Nanogels size, surface charge, stealth-coating, and responsive properties can be controlled through the synthesis process of the nanomaterials. These properties play a major role in drug delivery performance. For instance, Spencer et al. used an atom transfer radical polymerization (ATRP) synthesis approach to control the nanogel’s hydrodynamic size, pKa, volume swelling ratio, and the pH range over which volume swelling occurs. Three sets of nanogels were synthesized to investigate the effects of cationic monomer hydrophobicity, comonomer content, and crosslinking density on the responsive properties of the nanogels. The nanogels exhibited a volume swelling transition dependent on the hydrophobicity of the cationic monomer in the formulation. The hydrodynamic size was controlled by varying the volume fraction of the organic phase of the formulation between 2.5 and 7.5 vol%. A decrease in size was observed in formulations with lower organic phase volume. The overall size was kept under 150 nm. A co-stabilizer (n-hexadecane) was substituted as a fraction of the organic phase volume as a tool for optimizing size. As the volume fraction of n-hexadecane increased, nanogels decreased in both swollen and collapsed size and maintained low polydispersity indexes [181].

Self-assembled nanogels have gained popular attention in the ocular drug delivery field [182,183]. The physiological and anatomical complexity of the eye make it a highly protected organ, substantially limiting drug delivery despite the ability to treat topically. Thus, designing effective drug delivery systems to target specific areas of the eye is a major research challenge. Hydrogels enable sustained and controlled release of the drug and improve the drug’s bioavailability [184]. Liu et al. designed a biodegradable, microsphere-hydrogel for the controlled and extended release of ranibizumab. The PLGA microspheres were loaded with ranibizumab; consequently, these spheres were loaded to a poly(ethylene glycol)-co-(L-lactic acid) diacrylate/N-isopropylacrylamide (PEG-PLLA-DA/NIPAAm) hydrogel. Sustained release of ranibizumab was demonstrated for a period of 176 days [185]. Currently, there are formulations commercially available or in clinical trials aimed at treating glaucoma and bacterial conjunctivitis [186,187,188]. Shedden et al. evaluated the efficacy of timolol maleate ophthalmic gel-forming solution given once a day versus timolol solution given twice a day in a long-term trial. They demonstrated that the timolol maleate gel-forming solution is as effective as the timolol solution at reducing intra ocular pressure. Furthermore, the gel-forming solution provides the extra benefit of improving patient compliance by reducing amounts of administration per day [187]. A novel hybrid genipin-crosslinked dual-sensitive hydrogel was designed by Yibin et al. for the delivery baicalin (BN), an ophthalmic anti-inflammatory drug. The dual pH- and thermo-sensitive hydrogel was composed of carboxymethyl chitosan (CMCS) and poloxamer 407 (F127), and it was crosslinked with genipin (GP) (GP-CMCS/F127) with an overall size of less than 100 nm. The hydrogel release BN in a biphasic manner. The authors suggest that the burst release is beneficial as it may facilitate the rapid onset of drug initially. Comparing the release profiles between the loaded hydrogel and the eye drops, the hydrogels showed slower release, making this a great vehicle for delivering drug to treat cornea-based conditions [189]. Yu et al. designed a self-assembled dexamethasone-glycol chitosan conjugates for topical ophthalmic drug delivery with mean size of less than 300 nm. The release profile shows sustained release of dexamethasone for 48 h, with the release rate being controlled by the amount of glycol chitosan in the formulation. Due to their positive net charge, this delivery system could extend the pre-corneal retention, proving enhanced drug bioavailability [190]. Bao et al. designed a glycol chitosan/oxidized hyaluronic acid hydrogel for the dual ophthalmic delivery of dexamethasone and levofloxacin. The release profile showed that most of the loaded cargo of levofloxacin was released within the first 10 min of the release experiment; while dexamethasone was sustainably released, with an initial burst release of 18% of the loaded cargo within the same time frame. The in-vivo studies showed that the hydrogel films present anti-inflammatory properties via the marked regulation of various inflammatory cytokines, making this film suitable for treating postoperative endophthalmitis [191].

Moreover, hydrogels have obtained considerable attention in the oncology field. The use of nanotechnology has extensively increased due to the efficient and targeted delivery of chemotherapeutics. A suitable nanogel for oncology should have high drug loading efficiency, safely carry the drug, and release it in a controlled manner being sensitive to the environment at the target site [192]. Tan and Liu formed linoleic acid (LA) modified carboxymethyl chitosan (LCC) nanogels for the controlled release of Adriamycin (ADR), with an average particle size that was below 500 nm. The release rate was controlled through the degree of substitution of the LCC nanoparticles (number of linoleic acid groups per anhydroglucose unit). The higher the degree of substitution, the slower the release rate of ADR [193]. More recently, Gonzalez-Urias et al. designed a fluorescent nanogel with potential applications in theranostics. The multi-stimuli responsive nanogels (75 to 150 nm) were crosslinked and synthesized with fluorescein O,O′-diacrylate (FDA). The release rate of cisplatin (CDDP) and 5-fluorouracil (5-FU) was controlled through the pH of the release medium, with about 70% of the loaded drug released during the first 30 h. In-vitro studies showed cellular uptake after 4 h of incubation [194]. 

Hypoxia-responsive nanogels are at the forefront of oncology drug delivery. Peng et al. reported the synthesis and fabrication of a hypoxia-degradable zwitterionic poly(phosphorylcholine)-based (^H^PMPC) nanogel with an average size below 200 nm. Under hypoxic conditions, the doxorubicin loaded nanogels released about 86% of the loaded cargo through the degradation of the azobenzene bond. In-vitro studies showed that cells were successfully internalizing the nanoparticles [195]. Controlling drug delivery response through size modification of the nanogels is yet another technique used for cancer drug delivery. Choi et al. designed a degradable micro/nanomotor delivery system. The nano/micromotor is composed of a platinum (Pt) deposited complex of calcium carbonate and cucurbit[6]uril-conjugated hyaluronate (Pt/CaCO_3_@HA-CB[6]) of about 1 µm. At pH 6.5, the complex disintegrates by dissociation of CaCO_3_ and the encapsulated HA-CB[6], of approximately 300 nm, are released for cell uptake [196].

Hydrogels are categorized as the group of biocompatible, biodegradable and non-toxic drug delivery platforms. Tyliszczak et al. analyzed the toxicity levels on epidermal cells of chitosan hydrogels modified with gold nanoparticles as potential materials for drug delivery. Samples with low amounts of gold nanoparticles (1 to 3 mL) showed high levels of cell viability. Whereas samples with high amounts of gold nanoparticles (5 mL) exhibited cytotoxicity. Further studies indicated that the addition of the nanoparticles to the hydrogels had an impact in pore size reduction, which can be a potential tool to control the release rate of therapeutics [197]. Novel thermo-sensitive hydrogels based on PEG-poly(e-caprolactone-co-1,4,8-trioxa[4.6]spiro-9-undecanone) (PECT) are being developed for placitaxel delivery. Wang et al. are focusing on the toxicity and in-vivo biological effect of such hydrogels (3 to 5 μm pore size). The results showed that cell viability was around 100% with varying concentration of the triblock copolymer in the formulation. Furthermore, in-vivo studies showed that PECT-based hydrogels didn’t induce a mutagenic response under the conditions of the study [198].

### 4.3. Vesicles

**Figure 5:**
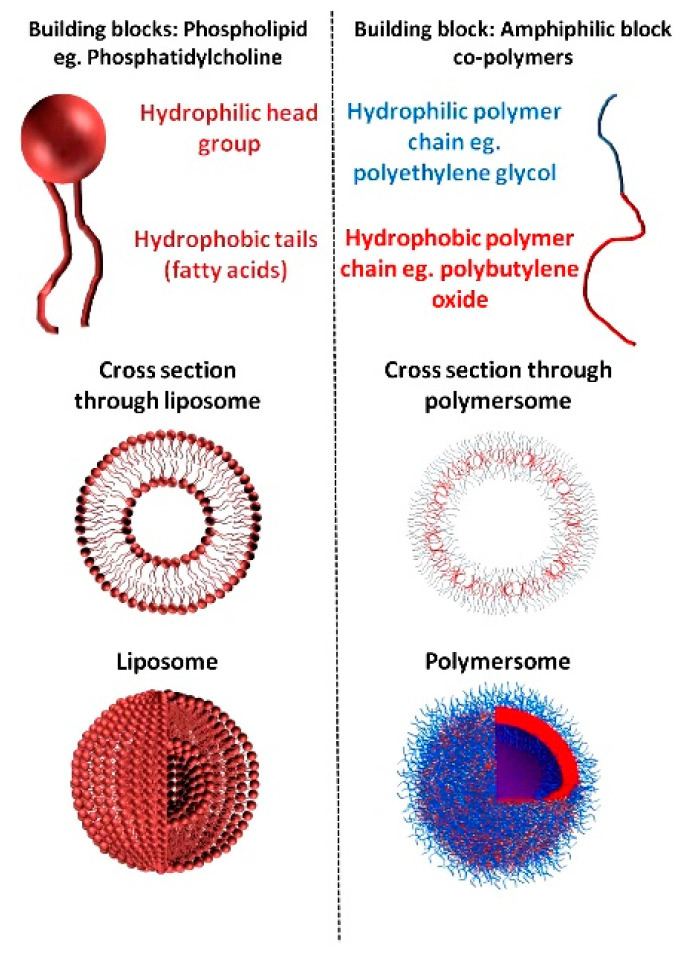
Difference Between Liposomes and Polymersomes. They are both self-assembled structures with different types of building blocks. Liposomes are made of phospholipids. While, polymersomes are made of block-copolymers (Reproduced with permission from [201]; Copyright Elsevier, 2020).

Vesicles are yet another type of self-assembled systems (Figure 3). Polymersomes and liposomes are self-assembled structures that are classified as vesicles due to their architecture. Vesicles are small, hollow, membrane-bound spheres composed of phospholipids (liposomes/vesicles) or block copolymers (polymersomes) (Figure 5). These structures are ideal for combination therapy since both hydrophobic and hydrophilic compounds can be delivered [48,199]. Hydrophilic compounds are encapsulated in the hollow core, while hydrophobic compounds are loaded within the bilayer. Their robust, stable, and tunable membranes, high loading capacity, and long blood circulation times further augment their value in the targeted drug delivery of a wide variety of therapeutics with tuned pharmacokinetics [16,17,48,200].

Liposomes were discovered in the early 1960s by Alec Bangham [202]. These structures were originally used as cell membrane models [203]. It was not until the 1970s when liposomes were first proposed as a simple drug delivery system [204]. Factors, such as the lipid composition, number of lipid bilayers, size, surface charge, non-polar chain length, degree of unsaturation, and the method of preparation, define liposome properties [205,206]. Their size varies from 50 nm to 1 um, depending on the number of lipid layers (lamellae). The main factors that affect vesicles’ half-life and drug encapsulation are size, the number of lipid layers, and the stabilizers (cholesterol). Liposomal functionality and structure can be controlled using cholesterol. It behaves as a stabilizer and helps increase the packing of phospholipid molecules [207], reduce bilayer permeability [208], and helps prevent liposomal aggregation [209]. Briuglia et al. (2015), investigated the most suitable amount of cholesterol in lipids to achieve stable and controlled drug release liposomes. They showed that a 70:30 molar ratio of phosphatidylcholine lipids to cholesterol achieved stability, as well as sustained and controlled the release of Atenolol and Quinine under sink conditions [210]. Lee et al. (2008), demonstrated that cholesterol in liposomes greatly increases their stability as well as the incorporation efficacy of the cargo (retinol). The stability of the liposomes and drug retainment was 90% increased using a 50:50 ratio of soybean phosphatidylcholine (PC) and cholesterol, and it remained after 10 days of storage [211].

The self-assembly of liposomes and polymersomes follow the same theory as previously described in Section 3. Polymersome self-assembly depends on the ratio of the hydrophilic part to the total mass of the copolymer. If the ratio is between 25 and 40%, the self-assembly of polymersomes is more favorable [212,213]. Polymersomes present better stability and versatility than liposomes, as they can be specifically designed and tailored so that their cargo can be released in response to common environmental changes, such as, pH [214,215,216], temperature [217], electrostatic forces, and ionic strength [200,218]. The main factors that control the size, stability, and shape of polymersomes are copolymer composition and molecular weight. However, there is typically still a large variation in the final hydrodynamic size distribution due to their self-assembly ratio. Lyoprotectants can further aid in controlling the size and structure of polymersomes during their fabrication and long-term storage, reducing the time and cost of separation techniques [213]. Byrne et al. (2016) studied the use of inulin and mannitol to preserve the stability and size distribution of polymersomes ideal for drug delivery (less than 200 nm). They demonstrated that the incorporation of mannitol significantly maintained polymersomes’ size diameter below 200 nm [213]. Advances in controlled polymer synthesis have enabled scientists to create polymersomes of various sizes. Ring opening polymerization reaction (ROP) is one of the easiest and most effective methods of designing amphiphilic polymers for degradable polymersomes. It allows for the addition of functional groups and heteroatoms into the backbone as well as hydrolytically cleavable ester and carbonate bonds. In 2020, Daubian et al. reported the synthesis of poly(ethylene oxide)-*b*-poly(2-(3-ethylheptyl)-2-oxaxoline) (PEO-*b*-PEHOx) polymersomes through cationic ROP. The hydrodynamic radius of the polymersomes varied from 19 to 178 nm, depending on the amount of PEHOx in the formulation [219].

Furthermore, polymersomes have been used in research as potential treatments for conditions like gliomas or lysosomal storage disease [53,220]. Lysosomal storage disorders like GM1 gangliosidosis are commonly treated with enzyme replacement therapy. However, this is a less efficient treatment since these are unable to cross the blood–brain barrier [221]. On the other hand, the use of polymersomes to deliver beta-galactosidase to the affected cells via the blood–brain barrier has shown to be very effective in feline models. It was demonstrated that polymersomes of less than 200 nm can protect the therapeutic cargo while crossing the blood–brain barrier, and can effectively target ligands to allow the transcytosis of the therapeutic to the affected neural cells [53].

In 2019, superparamagnetic, hybrid, self-assembled polymersomes (<200 nm) were designed for magnetic resonance imaging and magneto-chemotherapy. Maghemite nanoparticles were encapsulated within biodegradable block copolymer vesicles through nanoprecipitation and were loaded with doxorubicin hydrochloride. Controlled drug release was achieved for over 24 h [222]. Walvekar et al. developed novel hyaluronic acid—oleylamine (HA-OLA) conjugates as nano-drug carriers (<360 nm) for the delivery of antibiotic therapeutics like vanomycin (VCM). The release profile showed that VCM was slowly released from the polymersomes, with 57% of the loaded cargo released at 12 h. It took 72 h for the polymersomes to release 90% of the loaded cargo, while the same amount of free VCM was released in 24 h [223]. Japir et al. designed membrane-cross-linked polymersones with tumor pH-responsive and cross-linking density-mediated membrane permeability for controlled enzymatic drug delivery. The polymersomes (<500 nm) were prepared with PEG and methacrylate monomers containing piperidine or coumarin moieties (PEG-*b*-(PEMA_x_-co-CMA_y_). Glucose oxidase (GOD) and doxorubicin were encapsulated inside the self-assembled polymersomes. The release profile of doxuribicin shows that the release rate can be controlled by both the pH and the membrane-cross-linking density. About 15% of the loaded cargo was released at both pH 6.5 or 7.4 for 2 days. This showed that the membrane crosslinking kept the drug inside the polymersomes, preventing the leakage of drug at the physiological environment [224]. Zavvar et al. used PEG-PCL polymersomes encapsulated with gadolinium based quantum dots (QDs) and doxorubicin. Sustained release of doxorubicin was achieved for 10 days; the release rate was controlled by the pH of the release medium. These 100 nm polymersomes showed suitability as diagnostic and therapeutic tools [225].

Recent studies focus on the development of a new-generation of ultrasound-responsive polymersomes. Wei et al. developed a self-assembled polymersome made of poly(ethylene oxide)-*b*-poly(2(diethylamino)ethyl methacrylate)-stat-poly(methoxyethyl methacrylate) (PEO-B-P(DEA-stat-MEMA), with a diameter size of less than 600 nm, to evaluate intracellular delivery of doxorubicin. Cell viability was dose dependent, with over 86% viability for polymersome concentration between 62.5 and 500 ug/mL. In-vivo studies showed antitumor effect, suppressing tumor growth [226]. Zhou et al. developed light-responsive polymersomes with a charged surface for targeted delivery of doxorubicin. Their didodecyl substituted o-nitrobenzyl derivative polymersomes showed no significant toxicity to the cells after 48 h of incubation up to a concentration of 333 ug/mL; cell viability was above 90% [227]. Ghorbanizamani et al. used PEO-PCL polymersomes (<150 nm) to deliver a well-known antioxidant, L-glutathione (GSH), as a potential treatment for cancer. Cell viability studies showed that the polymersome formulation with and without drug was over 80%, regarding their safe use as nanocarriers [228].

Table 2 provides a list of FDA-approved biomaterials capable of forming polymersomes that have been used in controlled drug delivery.

## 5. Clinical Potential of Self-Assembled Structures in Drug Delivery

Conventional drug delivery systems are known for causing systemic side effects due to their nonspecific bio-distribution and uncontrollable drug release. Localizing medical treatments to the specific affected area to reduce systemic toxicity or side effects is one of the major goals to improve patient compliance. Hence, novel self-assembled drug delivery systems are being designed and developed to optimize drug release profiles to satisfy these unmet needs in the medical field. While it is important for these drug delivery vehicles to be non-toxic and have good biocompatibility, prolonged circulation time, controlled release and biodegradability, and cell specific targeting, these products must also be clinically efficient and cost effective. For example, the average cost and time needed to develop a new self-assembled drug delivery platform is about $20–50 million USD and 3–4 years. This is significantly lower than developing a new drug, which can take about $500 million USD and over 10 years [229]. Moreover, due to the efficacy of new self-assembled platforms, the global drug delivery market was valued at $31.96 billion USD in 2017, and it is expected to grow a CAGR of 14% in 2025 [230].

The majority of self-assembled systems that have been FDA-approved or are under clinical investigation use medicines that are already clinically-approved. This allows for a shorter approval process via 505 (b)^2^ pathway. In the past, many smart self-assembled drug delivery systems failed clinical trials because of the inability to demonstrate any significant improvements in efficacy, i.e., they failed to deliver an optimized drug dosage for a specified period of time compared to existing medications data. However, currently there are many nanoformulations under clinical development that are showing promising results for future commercial applicability [231]. Table 3 lists the self-assembled systems that are in clinical trials or have been FDA-approved.

## 6. Summary, Future Perspectives, and Tools

As the field advances, the use of self-assembled structures to improve therapeutic efficacy and efficiency will increase. Biodegradability, biocompatibility, improved drug stability, controlled and tailored payload release, reduced side effects, and improved patient compliance are many of the most important benefits of self-assembled nanocarriers. Self-organization, or self-assembly, at the nano- and macro-molecular scale provides an alternate and efficient way to deliver drugs compared to conventional drug delivery systems. The final properties of the self-assembled nanocarriers can be tailored at the monomer and molecular level, enabling specific properties and release profiles. Moreover, the final structures can be further modified for specifically targeted medical applications.

New tools for self-assembled drug delivery systems are emerging for the development of complex, multifunctional platforms [247,248]. Recently, nucleic acid-based cross-linkers have gained attention due to their ability to self-assemble into stable 3-dimensional structures using Watson–Crick base pairing. From this complementary base pairing, a multitude of methods exist to control the configuration of nucleic acid self-organized structures, including single-stranded “sticky ends”, duplex hybridization, and internal configurations such as G-quadruplex and hairpin formations [249]. Additionally, nucleic acids are able to act as targeting agents—through engineered aptamers—and drug payloads carriers [250,251]. Nucleic acid-based hydrogels have also shown the ability to control the release of proteins through selective sequence engineering [252,253]. Owing to this versatility, it is expected that nucleic acid-based hydrogels will become very prominent in the field in the future.

One of the most prominent challenges that self-assembled drug delivery platforms face is the optimization of performance of previously existing structures. Another significant challenge that self-assembled platforms face is the improvement of preclinical research to achieve clinical trial success. This can be achieved by designing new block copolymer monomers or nucleic acid “building blocks” with highly predictable behaviors. Future work should be focused on the improvement of drug delivery efficacy for proper clinical translation to ensure more of these self-assembled nanocarriers become FDA-approved and ultimately commercially available.

## Figures and Tables

**Table 1:** Examples of the different types of surfactants used in drug delivery.

Surfactant Formulation	Therapeutic(s) Delivered	Medical Application	Ref.
**Non-Ionic**			
SDC-PC (40 nm)	Ciprofloxacin	**	[34,35]
Sorbitan monostearate (Span 60) (77–84 nm)	Policarpine HCl	Ocular	[33,36]
Tween 80 (46 to 114 nm)	Curcumin	Brain	[37,38]
Brij 78 (90 to 120 nm)	Doxorubicin	Oncology	[39,40]
**Cationic**			
CKC (240 μm)	Dexamethasone 21-disodium phosphate	Ocular	[41,42]
Sodium alginate-HPMC	Cripofloxacin Hydrochloride	Ocular	[43]
DTAB (60–90 nm)	Meloxicam	Dermal Delivery	[44,45]
CPC (60–90 nm)	Meloxicam	Dermal Delivery	[44,46]
**Anionic**			
SLS (130–220 nm)	Propranolol HCl	**	[47,48]
ST (20 nm)	Propranolol HCl	**	[47,49]
SDS (110–250 nm)	**	Transdermal drug delivery	[50,51]
DA (400 nm)	(+)-catechin	Transdermal drug delivery	[52,53]
**Amphoteric**			
pDoAo	Oxytetracycline	**	[48,54]
LSB	**	Transdermal drug delivery	[49,55]
Lecithin (611 nm)	Doxorubicin Hydrochloride	**	[56]
PSBMA (230–290 nm)	Doxorubicin	Oncology	[57]

N-((4-sulfamoyphenyl)carbamothyol)stearamide (SDC-PC), Poly(ethylene glycol) sorbitan monooleate (Tween 80), Poly(ethylene glycol) octadecyl ether (Brij 78), Cetalkonium chloride (CKC), hydroxypropylmethylcellulose (HPMC), Dodecyltrimethylammonium bromide (DTAB), Cetylpyridinium chloride monohydrate (CPC), Sodium lauryl sulfate (SLS), Sodium taurocholate (ST), Sodium dodecylsulfate (SDS), Deoxycholic acid (DA), p-dodecyloxybenzyldimethylamine N-oxide (pDoAo), Lauryl sulfonate betaine (LSB), Poly(sulfobetaine methacrylate) (PSBMA). ** These papers did not discuss the drug released or the potential medical applications of the self-assembled system of interest.

**Table 2:** List of FDA approved biopolymers used in the formation of self-assembled structures for controlled delivery.

Formulation	Therapeutic(s) Delivered	Medical Application	Benefits	Ref.
**Micelles**				
PCL-*b*-PEG-*b*-PCL (10 nm)	Dexamethasone Docetaxel	Ocular Delivery Oncology	Extended Release	[35] [107]
PLGA-*b*-PEG-*b*-PLGA (77–84 nm)	US597@micelles	Oncology	Sustained oral formulation	[36]
PLA-*b*-PEG (<200 nm)	Rifampin	Bacterial infections	Micelle morphology and release profile controlled by the stereocomplex structure of PLA	[38]
Pluronics^®^ (<60 nm)	Genistein, paclitaxel and quercetin Hydrochorothiazide	Oncology Diuretic	Extended Release	[40] [91]
PGA-*b*-PAE (100–200 nm)	Cisplatin	Oncology	Improved drug loading with small sized micelles	[108]
PLL-*b*-DOCA-*b*-mPEG (<200 nm)	Curcumin	Oncology	Prolonged blood circulation time and provided successful biodistribution images	[109]
PEG-*b*-Pasp (22 to 60 nm)	Diminazene aceturate	**	Non-covalent interactions to form polyionic micelles	[110]
PLH-*b*-PEG (112 nm)	Paclitaxel	Oncology	Fast pH controlled drug release and cell internalization	[111]
PEI-*g*-PVP (142 nm)	Folic acid	**	Drug loaded through electrostatic interaction. Drug release rate moderated by pH	[112]
PDMAEMA-PCL (<150 nm)	siRNA and paclitaxel	Oncology	Co-delivery of drugs with different physicochemical properties	[113]
PEG-*b*-PLL-*b*-PLLeu (100–125 nm)	Docetaxel and siRNA-Bcl-2	Oncology	Cationic micelles for passive targeting of cancer cells	[114]
PIHCA-Tween80 (<320 nm)	Doxorubicin	Oncology	Spherical nanoparticles with high loading percentages	[115]
**Hydrogels**				
Pluronics^®^	Lidocaine	Topical Formulations	Release rate was controlled through the viscosity of the hydrogel	[42]
Sodium alginate-HPMC	Cripofloxacin Hydrochloride	Ocular	pH responsive release system	[43]
PCL-*b*-PEG-*b*-PCL	Dexamethasone Insulin	** Glucose control	Extended Release	[116] [45]
PEO-*b*-PHB-*b*-PEO	FITC-Dextran	**	Extended Release	[46]
PLGA-*b*-PEG-*b*-PLGA	Levonorgestrel DNA	Birth Control Gene therapy	Extended Release	[117] [118]
OncoGel^TM^	Paclitaxel	Solid tumors	Extended Release	[119]
PAH/Chitosan	Ciprofloxacin hydrochlorine monohydrate	**	Release of hydrophilic and/or unstable agents	[120]
**Vesicles (Polymersomes/Liposomes)**				
PLA-*b*-PEG-*b*-PLA (200–300 nm)	Atorvastatin and lisinopril	Oncology	High encapsulation efficiency of hydrophobic and hydrophilic drugs	[48]
mPEG-*b*-(PPLG-*g*-MSA) * (20 nm)	Doxorubicin Hydrochloride	**	Micelles formed through electrostatic interactions	[49]
PLL-*b*-PBLG-*b*-PEO (<300 nm)	Doxorubicin and Paclitaxel	Pancreatic cancer	Temperature- and pH responsive release	[51]
PEG-*b*-PLA (<200 nm)	Active beta-galactosidase	Enzyme Replacement Therapy	pH responsive release system	[53]
PS (100 nm)	Arsenic Trioxide	Glioblastoma Multiform (GBM)	pH responsive system	[121]
Lecithin/Chitosan (240 nm–1 μm)	Tamoxifen citrate	Oncology	Oral administration. Relese rate controlled by enzymatic degradation	[122]

Poly(ethylene glycol) (PEG), poly(lactic acid) (PLA), poly(lactic-co-glycolic acid) (PLGA), poly(propylene oxide) (PPO), poly(caprolactone) (PCL), Pluronics^®^ (PPO-PEO), poly(γ-L-glutamic acid) (PGA), poly(L-phenylalanine ethyl ester) (PAE), poly(L-Lysine) (PLL), methyl-PEG (mPEG), poly(aspartamic acid) (PasP), poly(L-histidine) (PLH), poly(ethylene amine) (PEI), poly(N-vinylpyrrolidone) (PVP), poly(L-Leucine) (PLLeu), deoxycholic acid (DOCA), hydroxy propyl methyl cellulose (HPMC), poly(hydroxy butyrate) (PHB), poly(ethylene oxide) (PEO), poly(γ-benzyl-L-glutamate) (PBLG), phosphatidylserine (PS), poly(isohexyl-cyanoacrylate) (PIHCA), poly(allylamine hydrochlorine) (PAH). * poly(γ-propargyl) (PP) is not FDA-approved. ** These papers did not discuss potential medical applications of the self-assembled system of interest.

**Table 3:** Commercially-available products and clinical trials utilizing self-assembled structures.

Formulation	Therapeutic(s)	Medical Application	Clinical Phase	Ref.
**Micelles**				
PEG-pAsp	Paclitaxel	Advanced stomach cancer	II	[232]
PEG-*b*-pAsp	Doxorubicin	Pancreatic and colorectal cancer	II	[233]
Genexol™-PM (20–50 nm)	mPEG-PLGA-Paclitaxel	Breast cancer	IV	[234]
Adynovate	PEGylated factor VII	Hemophilia A	FDA Approved, 2015	[16] [235]
Estrasorb^TM^	Estradiol	Menapause hormone Therapy	FDA Approved 2003	[178]
Cimzia^®^	PEGylated antibody fragment	Chron’s disease, rheumatoid arthritis, psoriasis	FDA Approved 2008–2013	[178]
Mircrea^®^	Erythropoiesis-stimulating agent	Anemica with chronic renal failure	FDA Approved 2007	[178]
Plegridy^®^	PEGylated IFNbeta-1a	Multiple Sclerosis	FDA Approved 2014	[178]
**Polymersomes/Liposomes**				
Doxil^®^ (200–500 nm)	Doxorubicin	Ovarian cancer AIDS-related Kaposis’s sarcoma; breast cancer	FDA approved, 1995	[236] [237]
Amphotech^®^	Amphotericin	Fungal infection	FDA approved, 1996	[237]
Myocet	Doxorubicin	Metastatic Breast Cancer		[238]
Marquibo^®^	Vincristine	Philadelphia chromosome-negative (Ph-) Acute lymphoblastic leukemia	FDA approved, 2012	[239]
ThermoDox (50 to 200 nm)	Doxorubicin	Breast cancer, primary liver cancer	II and III	[240]
Vyxeos^TM^	Daunorubicin and Cytarabine	Acute myeloid leukemia (AML), AML with myelodysplasia-related changes	FDA approved, 2017	[204,241]
Arikayce	Amikacin	Chronic lung infections	I, II and III	[242]
Lipoquin	Ciprofloxacin	Cystic fibrosis (CF) and Non-CF bronchiectasis	II	[231]
HER2-targeted MM302	Doxorubicin	HER2-positive breast cancer	I	[231]
ThermoDox^®^	Doxorubicin	Cancer	III	[243]
Onivyde	Irinotecan	Pancreatic Cancer	FDA Approved 2015	[16]
**Hydrogels**				
OncoGel^TM^	Paclitaxel	Solid tumors	I and II	[244]
Pluronics^®^	Doxorubicin	Advanced esophageal adenocarcinoma	III	[58]
Somatuline^®^	Lanreotide	Acromegaly	**	[245]
SpaceOAR^®^	PEGylated	Prostate cancer radiotherapy tissue protection	FDA Approved 2010, 2015	[246]
Vantas^®^	Histrelin acetate and gonadotropin releasing hormone	Prostate cancer	FDA Approved 2004, 2005	[246]
Radiesse^®^	Hydroxylapatite	Production of collagen	FDA Approved 2015	[246]

** The clinical trial phase was not discussed.
